# Validating the virtual: a deep dive into ultrasound simulator metrics in otorhinolaryngology

**DOI:** 10.1007/s00405-023-08421-y

**Published:** 2024-01-04

**Authors:** Anne Line Risgaard, Iben Bang Andersen, Mikkel Lønborg Friis, Martin Grønnebæk Tolsgaard, Christian Sander Danstrup

**Affiliations:** 1 NordSim, Centre for Skills Training and Simulation, Aalborg University Hospital, Aalborg, Denmark; 2 Department of Otorhinolaryngology – Head and Neck Surgery, Aalborg University Hospital, Aalborg, Denmark; 3 Department of Clinical Medicine, Aalborg University, Aalborg, Denmark; 4 Copenhagen Academy for Medical Education and Simulation (CAMES) Rigshospitalet and Center for Fetal Medicine, Copenhagen University Hospital Rigshospitalet, Copenhagen, Denmark

**Keywords:** Validity evidence, Assessment of learning, Head and neck ultrasound, Ultrasonography, Simulation-based medical education, Diagnostic accuracy

## Abstract

**Purpose:**

This study aimed to assess the validity of simulation-based assessment of ultrasound skills for thyroid ultrasound.

**Methods:**

The study collected validity evidence for simulation-based ultrasound assessment of thyroid ultrasound skills. Experts (*n* = 8) and novices (*n* = 21) completed a test containing two tasks and four cases on a virtual reality ultrasound simulator (U/S Mentor's Neck Ultrasound Module). Validity evidence was collected and structured according to Messick’s validity framework. The assessments being evaluated included built-in simulator metrics and expert-based evaluations using the Objective Structured Assessment of Ultrasound Skills (OSAUS) scale.

**Results:**

Out of 64 built-in simulator metrics, 9 (14.1%) exhibited validity evidence. The internal consistency of these metrics was strong (Cronbach’s *α* = 0.805) with high test–retest reliability (intraclass correlation coefficient = 0.911). Novices achieved an average score of 41.9% (SD = 24.3) of the maximum, contrasting with experts at 81.9% (SD = 16.7). Time comparisons indicated minor differences between experts (median: 359 s) and novices (median: 376.5 s). All OSAUS items differed significantly between the two groups. The correlation between correctly entered clinical findings and the OSAUS scores was 0.748 (*p* < 0.001). The correlation between correctly entered clinical findings and the metric scores was 0.801 (*p* < 0.001).

**Conclusion:**

While simulation-based training is promising, only 14% of built-in simulator metrics could discriminate between novices and ultrasound experts. Already-established competency frameworks such as OSAUS provided strong validity evidence for the assessment of otorhinolaryngology ultrasound competence.

**Supplementary Information:**

The online version contains supplementary material available at 10.1007/s00405-023-08421-y.

## Introduction

Point-of-care ultrasound has gained prominence across medical disciplines, particularly in otorhinolaryngology (ORL). The superficial anatomical positioning of the head and neck structures allows for high-resolution ultrasound (US) imaging, making it a preferred diagnostic tool. Pathology such as lymph nodes, salivary gland tumors and thyroid nodules can be well-described. US is an inexpensive and fast examination enabling bedside evaluation in the outpatient clinic and in private ORL practice. Furthermore, US can be used to guide fine needle aspiration biopsies to improve cytology sampling if neck pathology is found [1, 2].

In the evaluation of thyroid nodules, US has become an important tool. Nodules can be found in both asymptomatic and symptomatic patients and a thorough and systematic US approach is essential for an adequate evaluation [7]. Within the later years, tools such as the European Thyroid Imaging and Reporting Data System (EU-TIRADS) have become an important tool in the evaluation of the thyroid. However, this tool and the success of thyroid imaging are largely contingent on the operator's proficiency, which underscores the significance of effective training [4].

The highly operator-dependent nature of US imaging presents potential risks. Inaccurate diagnostics not only compromise patient safety but also emphasize the criticality of structured training, adherence to standardized guidelines, and periodic evaluations of operators [5–7].

The training prerequisites vary across regions. The American Institute of Ultrasound in Medicine mandates that novices complete 100 supervised head and neck US examinations before they are deemed competent for unsupervised practice [6]. Conversely, the European Federation for Ultrasound in Medicine and Biology suggests 300 supervised scans [8]. Yet, there is very limited evidence to support that volume is a predictor of diagnostic competence. In one study involving a large group of French sonographers, volume was found to be a necessary but insufficient factor for the development of US competence [9].

Instead, there is a movement toward the concept of mastery-learning, which involves assessment and training until a pre-defined mastery-learning level has been achieved. Whereas this approach ensures that trainees only finish after receiving an adequate level of training, it requires methods for reliable and valid assessment of competence [10]. In addition, traditional clinical training requires substantial faculty resources for bedside teaching and assessment. Instead, simulation-based US training has emerged as a promising avenue. By providing a controlled, risk-free environment, it allows trainees to refine their skills and gain confidence without impacting patient well-being [3, 11, 12]. Many of these simulators allow automated assessments of competence, however, with varying levels of validity evidence supporting their use [10, 13].

Anchored in these developments, this study aims to evaluate validity evidence for the assessment of thyroid US. Our study examines what evidence supports the use of automated built-in simulator assessments as well as the use of generic expert-based assessments of competence. We further contribute to the literature by examining how these assessments relate to diagnostic accuracy, identifying the best methods for assessing skills necessary for accurate diagnostics.

## Methods

### Study design and setting

The study was conducted from February to April 2023 at the Department of Otorhinolaryngology—Head and Neck surgery and NordSim—Centre for Skills Training and Simulation, Aalborg University Hospital, Aalborg, Denmark.

The primary focus was to assess the validity evidence of simulator metrics used in simulation-based US examinations of the thyroid.

Experts were selected from ORL specialists at Aalborg University Hospital with proficiency in US usage. Novices were medical students from the University of Aalborg, in their 8th to 10th semesters, having no prior experience with thyroid US.

### Equipment and simulator module

**Fig. 1:**
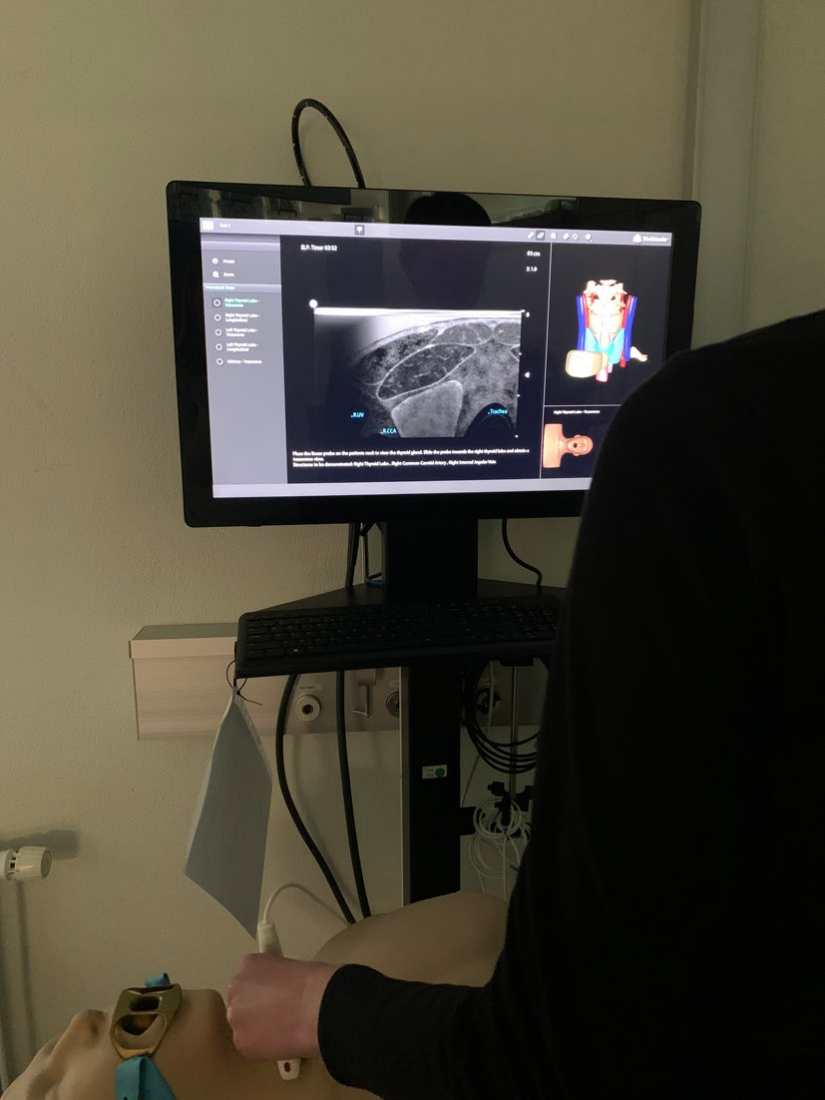
Picture of Task 1 in the neck ultrasound module of the U/S mentor

The study utilized the Neck Ultrasound Module of the U/S Mentor (Simbionix Ltd, Airport City, Israel). This simulator comprises a mannequin simulating a real patient, a monitor displaying simulated US images corresponding to probe movements on the mannequin, and a linear probe. Operators could adjust various parameters, such as depth of field, focus, and gain. The module encompassed two tasks and seven cases. Figure 1 displays a picture of the mannequin and monitor of the U/S Mentor.

### Validity evidence collection

To investigate validity evidence of the simulator metrics, Messick’s validity framework was used [14]. Furthermore, an assessment was conducted using the generic validated Objective Structured Assessment of Ultrasound Skills (OSAUS) scale [11].

Content evidence: Two medical educators and one ORL specialist identified relevant tasks and cases based on their significance in ORL [15].

Response process: Participants received standardized instructions regarding the simulator and its tasks and cases. The instructions, based on a predetermined protocol, covered accessing tasks, completing cases, and using specific simulator functions. No time limit was set for the test, and feedback was withheld. Technical assistance was available, but guidance on task completion was not provided.

Relations to other variables: Metrics able to discriminate between expert and novice levels were deemed to possess validity evidence when demonstrating a statistically significant difference (*p* < 0.05) between expert and novice performance.

Internal structure: The study evaluated test/retest reliability via the intraclass correlation coefficient and assessed metric internal consistency using Cronbach’s α. Novices took the test twice to evaluate test–retest reliability.

Consequences: To explore the test consequences and establish a pass/fail level of the test, the contrasting groups’ method was used. This method evaluated procedure performance across different expertise levels, determining a threshold for pass/fail, which enabled the calculation of false positives and negatives [16].

### Objective structured assessment of ultrasound skills evaluations

In addition to the built-in simulator metrics, all performances of Case 5 were also evaluated using OSAUS by one ORL specialist, who is an expert in head and neck US. Performances were evaluated through blinded video-review of the participants’ performances. The OSAUS scale comprises seven US-related items of which relevant items were selected by two simulation experts [11].

### Ethics and approval

This study was conducted in compliance with the General Data Protection Regulation and is a part of North Denmark Region’s record of processing activities (F2023-016).

Written informed consent was obtained from all participants before beginning the study. All methods were carried out in accordance with guidelines and regulations regarding Good Clinical Practice.

Due to the study dealing with medical education and does not involve new information regarding the emergence, prevention, diagnostics, and treatment of new diseases, ethical approval was not required according to The North Denmark Region Committee on Health Research Ethics (2023–000206).

### Statistical analysis

**Fig. 2:**
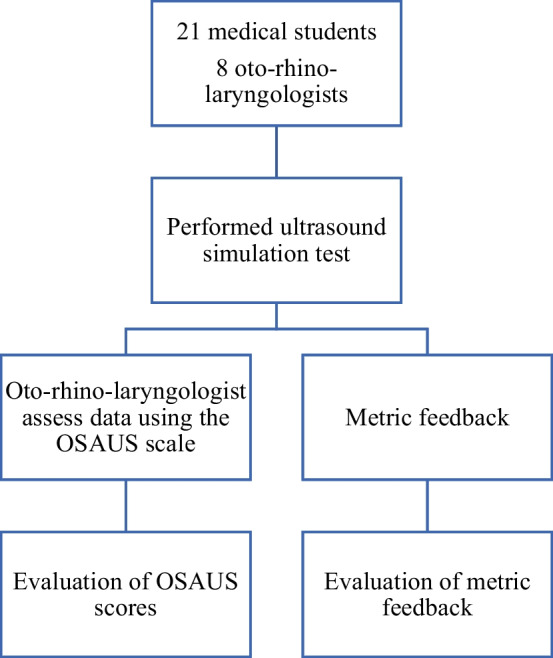
Flowchart of the process. OSAUS = objective structured assessment of ultrasound skills

The study's analytical approach is outlined in Fig. 2. Data were processed using SPSS (IBM Corp. Released 2021. IBM SPSS Statistics for Macintosh, Version 28.0. Armonk, NY: IBM Corp) with a statistical significance level set at *p* < 0.05. Metrics showing significant differences were graded as pass (1) or fail (0). The Student’s *t *test determined if there was a significant difference in metric feedback between novices and experts. Internal consistency was evaluated using Cronbach’s α, while the intraclass correlation coefficient assessed the test–retest reliability for novices. Mann–Whitney *U* tests were used for comparing OSAUS scores from novices and experts.

**Fig. 3:**
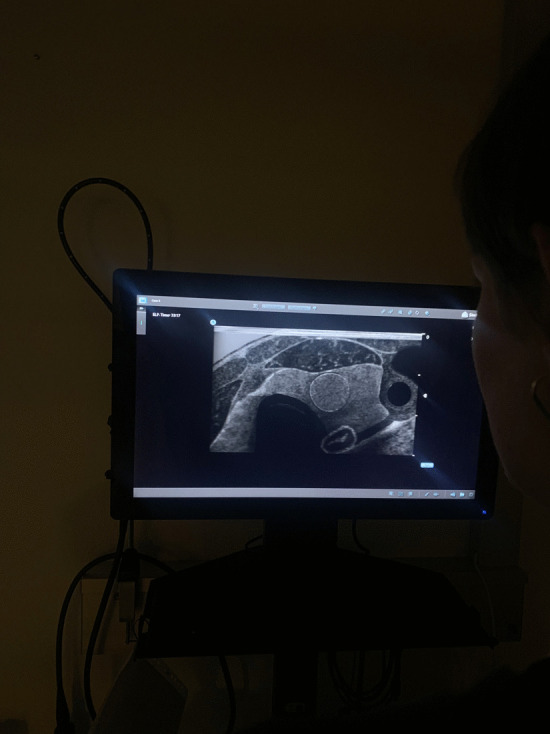
Picture of Case 5 in the neck ultrasound module of the U/S mentor. Measurements of the nodule were 0.99 cm in both length and width

To evaluate the correlation between correctly entered clinical findings and both OSAUS and metric scores in Case 5, two ORL experts determined the correct options in clinical findings. The correlations were determined using Spearman’s *ρ*. Figure 3 displays a picture of a thyroid with a nodule in Case 5 of the U/S Mentor.

## Results

This study included 8 experts and 21 novices. The basic demographics of the participants are shown in Table 1**.**

**Table 1:** Basic demographics of the 21 novices and eight experts in this study

Surveyed information	Novices (n = 21)	Experts (n = 8)
Women, *n*	16	2
Men, *n*	5	6
Median age (range)	24 (22–32)	43 (37–58)
Median years of experience (range)	0	9 (5–30)

### Simulator metrics

Content evidence: Two tasks and four cases deemed relevant for our investigation regarding thyroid US examination why they were selected, which included 64 simulator built-in metrics. Description of selected tasks and cases are displayed in Supplementary material 1.

Response process: Time spent during the tests was compared between the experts (median of 359 s, range: 98–959 s) and novices (median of 376.5 s, range: 166–758 s).

Relations to other variables: Only nine out of the initial 64 metrics (14.1%) were able to discriminate between novices and experts (*p* < 0.05). Table 2 displays the distribution of the in-built metrics that were able to discriminate and those that were unable to discriminate between novices and experts, respectively.

**Table 2:** Displays the distribution of the discriminating and non-discriminating metrics devoted into different categories

Metric	Discriminating	Non-discriminating
Time used	0	13
Clinical findings	5	11
Standard views	3	12
Measurements	1	19
Total	9	55

Out of the nine metrics that discriminated between different levels of competence, none were related to time used, 55.6% were related to the evaluation of clinical findings, 33.3% were related to standard view presentation, and 11.1% were related to correctness of measurements.

Supplementary material 2 displays all selected metrics of the Neck Module of the U/S Mentor. Out of the 55 metrics that did not discriminate between different levels of competence, 23.6% were related to time used, 20% were related to evaluation of clinical findings, 21.8% were related to standard view presentation and 34.5% were related to correctness of measurements.

### Internal structure

The internal consistency of the metrics with validity evidence was assessed with the Cronbach’s *α* being 0.805. The test/retest reliability was calculated based on mean-rating (*k* = 21), absolute-agreement, two-way mixed effects model leading to an intraclass correlation coefficient of 0.911.

### Consequences

**Fig. 4:**
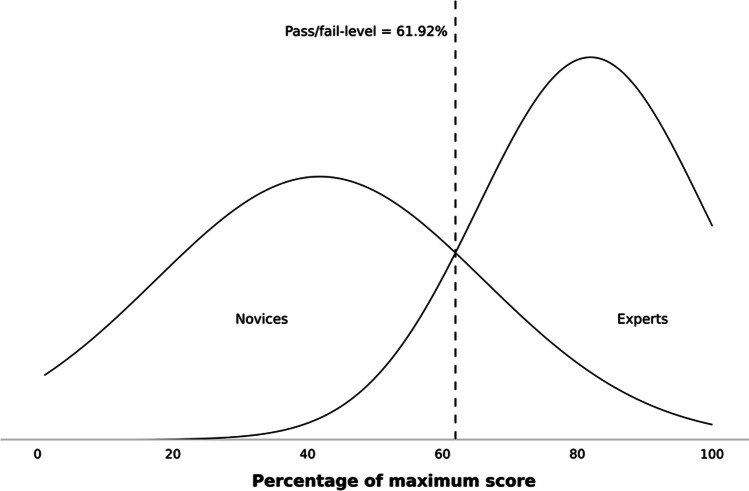
Pass/fail level. The pass/fail-level indicates that one competent operator failed the test and five incompetent operators passed the test. The *x*-axis demonstrates the percentage of the maximum simulator sum score. The graph is based on the 9/64 metrics possessing validity evidence

The novice group had a mean sum score of 41.9% (SD = 24.3) of the maximum score (sum scores on the nine discriminating metrics). The expert group had a mean sum score of 81.9% (SD = 16.7) of the maximum score. Using the contrasting groups’ method, a pass/fail level of 61.92% of the maximum test score was found. The consequences were that one competent operator failed and five incompetent operators passed, see Fig. 4.

### Objective structured assessment of ultrasound skills evaluations

Four items, including “Applied knowledge of ultrasound equipment”, “Image optimization”, “systematic examination” and “Interpretation of images”, were relevant for our investigation. All results regarding OSAUS are based on performances in Case 5.

**Fig. 5:**
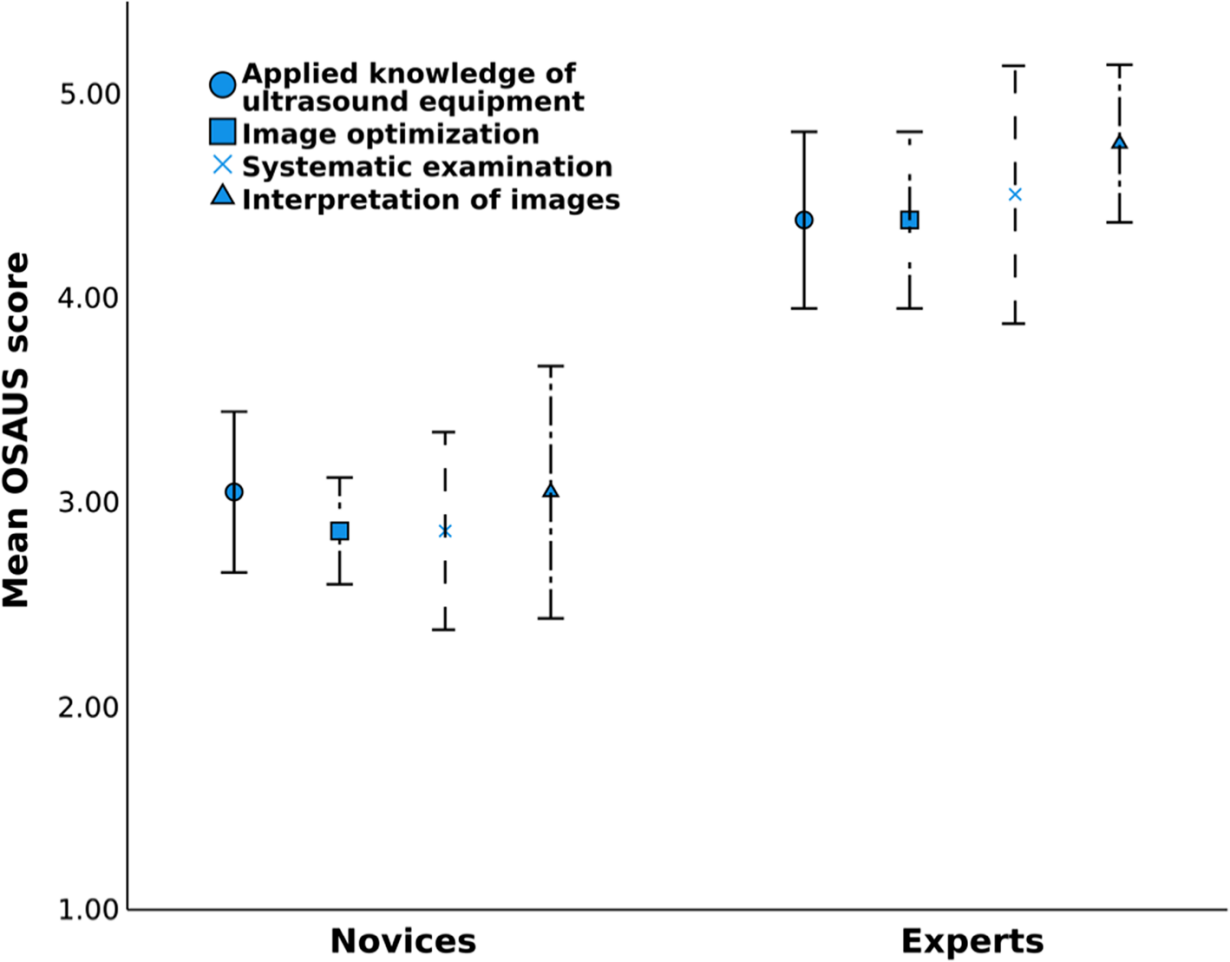
Distribution of mean scores on the four selected OSAUS items with a 95% confidence interval. OSAUS = Objective Structured Assessment of Ultrasound Skills

All the included items of OSAUS received a score that was statistically significant between the novice (median 3; range 1.0–5.0) and expert group (median 5; range 3.0–5.0), *U* = 294, *p* < 0.001, see Fig. 5.

Table 3 displays the distribution of the items with statistically significant difference between the group of novices and the group of experts.

**Table 3:** Distribution of items with statistically significant difference between the group of novices and the group of experts

OSAUS item	Novices (*n* = 21) Mean rank	Experts (*n* = 8) Mean rank	*Z* value
Applied knowledge of ultrasound equipment	12.1	22.5	– 3.38†
Image optimization	11.4	24.5	– 4.5†
Systematic examination	12.6	21.2	– 2.8†
Interpretation of images	12.3	22	– 3.16*

*p < 0.05, † *p* < 0.001. OSAUS = Objective Structured Assessment of Ultrasound Skills

The pass/fail level was 76.1% with a risk of 10.4% false positives and 4.9% false negatives. The expert group passed 97% of the OSAUS items, only having one expert failing the item "Systematic examination". The novice group passed 25% of the OSAUS items. The item "Interpretation of images" had the highest number of novices passing (33.3%) and the item "Image optimization" had the lowest number of novices passing (9.52%).

Correlations between clinical findings and scores.

Two experts determined correctly entered clinical findings in Case 5, see Supplementary material 3.

The correlation between correctly entered clinical findings and the OSAUS score showed a correlation of 0.748 (*p* < 0.001). Similarly, the correlation between correctly entered clinical findings and the metric score was 0.801 (*p* < 0.001). The correlations were based on Case 5.

## Discussion

The evolution of medical training has been marked by an increasing reliance on technology, particularly simulators, to offer trainees a risk-free environment to practice their skills [17]. Our study’s exploration into the validity of simulation-based assessment of US skills offers insights that hold significant implications for the future of medical education, especially in the realm of ORL.

The consistency of our findings with previous research, indicating a low proportion of simulator metrics with validity evidence, raises pressing questions [10, 18]. Are current simulator metrics sufficiently capturing the complexities of real-world US operations? According to our findings, this does not seem to be the case. Yet, our study further accentuates the potential of expert-driven tools, such as the OSAUS scale, which demonstrated a broader spectrum of discriminating features compared to simulator metrics. This disparity underscores the potential benefits of a multi-faceted assessment approach. Instead of relying solely on simulator metrics, combining them with generic assessments such as the OSAUS scale might offer a more holistic view of a learner’s proficiency. This will represent the first important step toward mastery-learning of thyroid US in ORL.

The blinding of the ORL specialist was a strength of our methodology. Yet, a potential limitation was the absence of multiple evaluators, which could have offered insights into inter-rater reliability. However, a previous generalizability study demonstrated that a sufficiently high reliability can be achieved with a single rater for multiple assessments using OSAUS (five per rater) with substantially fewer needed for two raters [19]. In addition, while our study population was diverse, extending the research to include participants from different regions or training backgrounds might offer a more comprehensive view of the topic.

The positive correlation between both scores and the accuracy of clinical findings, while promising, is merely the tip of the iceberg. It points to a myriad of potential research avenues, from exploring the underlying factors driving this correlation to assessing its implications for patient outcomes. However, the diagnostic cases may lack some essential nuances. Based on the traits of the thyroid nodule (Fig. 3), the participants were asked to state the suspected malignancy. The nodule was measured to be 0.99 cm and using EU-TRIADS, the two experts in this study independently scored the nodule as EU-TIRADS 3 [20]. The risk of malignancy of EU-TIRADS 3 nodules has been described to be 2–4% [4]. In the presented case, “None” was chosen as the most correct of the presented options despite the described discrepancy. It is worthwhile to explore whether such questions of clinical findings may cause difficulties for the participants, particularly for those who are less experienced in the field.

Correlation between US skills and diagnostic competence has not been shown in the context of thyroid US before, and further studies are needed to dive deeper into mapping which diagnoses are difficult and should be trained more as well as into how different aspects of US skills impact diagnostic performance in the clinical setting [12]. Our study did not explore the clinical value or cost-effectiveness of initial simulation-based training—only that we may assess and monitor the development of skills in the simulated setting—and this is an important subject for future research to critically evaluate how future ORL are most effectively trained in thyroid US.

## Conclusion

Only 14% of built-in simulator metrics could discriminate between novices and experts. Yet, relying on already-established competency frameworks such as OSAUS provided strong validity evidence for the assessment of thyroid US competence. Future work should explore the clinical impact of simulation-based mastery learning, including its cost-effectiveness compared with less costly alternatives, such as apprenticeship learning.

### Supplementary Information

Below is the link to the electronic supplementary material.

Supplementary file1 (DOCX 28 KB)

## Data Availability

Datasets are available from the corresponding author upon reasonable request.
