# Supplementary material for: Validating the virtual: a deep dive into ultrasound simulator metrics in otorhinolaryngology
Source: Eur Arch Otorhinolaryngol. 2024 Jan 4;281(4):1905–11. doi: 10.1007/s00405-023-08421-y (PMC10942893; doi:10.1007/s00405-023-08421-y)
Supplement: Supplementary file 1 — Supplementary file1 (DOCX 28 KB) [file 405_2023_8421_MOESM1_ESM.docx]

**Supplementary material 1**

| Task 1 |
| --- |
| Obtain a transverse view of the right thyroid lobe |
| Obtain a longitudinal view of the right thyroid lobe |
| Obtain a transverse view of the left thyroid lobe |
| Obtain a longitudinal view of the left thyroid lobe |
| Obtain a transverse view of the isthmus |
| Task 2 |
| Obtain a transverse view of the right thyroid lobe and measure: |
| Right thyroid lobe width |
| Right thyroid lobe depth |
| Obtain a longitudinal view of the right thyroid lobe and measure: |
| Right thyroid lobe length |
| Obtain a transverse view of the left thyroid lobe and measure: |
| Left thyroid lobe width |
| Left thyroid lobe depth |
| Obtain a longitudinal view of the left thyroid lobe and measure: |
| Left thyroid lobe length |
| Obtain a transverse view of the isthmus and measure: |
| Isthmus transverse |
| Isthmus anteroposterior |
| Case 4 |
| Obtain a transverse view of the right thyroid lobe and measure: |
| Right thyroid lobe width |
| Right thyroid lobe depth |
| Obtain a longitudinal view of the right thyroid lobe and measure: |
| Right thyroid lobe length |
| Obtain a transverse view of the left thyroid lobe and measure: |
| Left thyroid lobe width |
| Left thyroid lobe depth |
| Obtain a longitudinal view of the left thyroid lobe and measure: |
| Left thyroid lobe length |
| Obtain a transverse view of the isthmus and measure: |
| Isthmus transverse |
| Isthmus anteroposterior |
| Enter “Clinical findings” and enter the findings in the different subjects: |
| Presence of thyroid nodule |
| Internal content of the nodule |
| Shape of the nodule |
| Margin of the nodule |
| Calcifications in the nodule |
| Suspected malignancy of the nodule |
| Case 5 |
| Obtain a transverse view of the right thyroid lobe and measure: |
| Right thyroid lobe width |
| Right thyroid lobe depth |
| Obtain a longitudinal view of the right thyroid lobe and measure: |
| Right thyroid lobe length |
| Obtain a transverse view of the left thyroid lobe and measure: |
| Left thyroid lobe width |
| Left thyroid lobe depth |
| Obtain a longitudinal view of the left thyroid lobe and measure: |
| Left thyroid lobe length |
| Obtain a transverse view of the isthmus and measure: |
| Isthmus transverse |
| Isthmus anteroposterior |
| Enter “Clinical findings” and enter the findings in the different subjects: |
| Presence of thyroid nodule |
| Internal content of the nodule |
| Shape of the nodule |
| Margin of the nodule |
| Calcifications in the nodule |
| Suspected malignancy of the nodule |
| Case 6 |
| Obtain a transverse view of the right thyroid lobe and measure: |
| Right thyroid lobe width |
| Right thyroid lobe depth |
| Obtain a longitudinal view of the right thyroid lobe and measure: |
| Right thyroid lobe length |
| Obtain a transverse view of the left thyroid lobe and measure: |
| Left thyroid lobe width |
| Left thyroid lobe depth |
| Obtain a longitudinal view of the left thyroid lobe and measure: |
| Left thyroid lobe length |
| Obtain a transverse view of the isthmus and measure: |
| Isthmus transverse |
| Isthmus anteroposterior |
| Enter “Clinical findings” and enter the findings in the different subjects: |
| Presence of thyroid nodule |
| Internal content of the nodule |
| Shape of the nodule |
| Margin of the nodule |
| Calcifications in the nodule |
| Suspected malignancy of the nodule |
| Case 7 |
| Obtain a transverse view of the right thyroid lobe and measure: |
| Right thyroid lobe width |
| Right thyroid lobe depth |
| Obtain a longitudinal view of the right thyroid lobe and measure: |
| Right thyroid lobe length |
| Obtain a transverse view of the left thyroid lobe and measure: |
| Left thyroid lobe width |
| Left thyroid lobe depth |
| Obtain a longitudinal view of the left thyroid lobe and measure: |
| Left thyroid lobe length |
| Obtain a transverse view of the isthmus and measure: |
| Isthmus transverse |
| Isthmus anteroposterior |
| Enter “Clinical findings” and enter the findings in the different subjects: |
| Presence of thyroid nodule |
| Internal content of the nodule |
| Shape of the nodule |
| Margin of the nodule |
| Calcifications in the nodule |
| Suspected malignancy of the nodule |

Description of included tasks and cases of the Neck Module of the U/S Mentor

**Supplementary material 2**

| **Task 1** |  |  |
| --- | --- | --- |
| Total task time | p = 0.173 |  |
| Average time per view | p = 0.107 |  |
| **Total time with ultrasound image** | **p = 0.021** |  |
| Optimally acquired standard views | p = 0.411 |  |
| Partially acquired standard views | p = 0.411 |  |
| **Task 2** |  |  |
| Total task time | p = 0.610 |  |
| **Total time with ultrasound image** | **p < 0.001** |  |
| Optimally acquired standard views | p = 0.602 |  |
| Partially acquired standard views | p = 0.602 |  |
| Measurements: Right thyroid lobe width | p = 0.900 |  |
| Measurements: Right thyroid lobe depth | p = 0.484 |  |
| Measurements: Right thyroid lobe length | p = 0.293 |  |
| **Measurements: Left thyroid lobe width** | **p = 0.017** |  |
| Measurements: Left thyroid lobe depth | p = 0.711 |  |
| **Measurements: Left thyroid lobe length** | **p = 0.012** |  |
| Measurements: Isthmus transverse | p = 0.282 |  |
| Measurements: Isthmus anterior posterior | p = 0.820 |  |
| Optimally measured | p = 0.754 |  |
| Fairly measured | p = 0.861 |  |
| Inaccurately measured | p = 0.197 |  |
| **Case 4** |  |  |
| Total procedure time | p = 0.056 |  |
| Total time with ultrasound image | p = 0.358 |  |
| Clinical findings: Thyroid nodule | p = 0.139 |  |
| Acquired standard views | p = 0.085 |  |
| Skipped standard views | p = 0.411 |  |
| Unrecognized views | p = 0.764 |  |
| Measurements: Right thyroid lobe width | p = 0.806 |  |
| Measurements: Right thyroid lobe depth | p = 0.655 |  |
| Measurements: Right thyroid lobe length | p = 0.483 |  |
| Measurements: Left thyroid lobe width | p = 0.444 |  |
| Measurements: Left thyroid lobe depth | p = 0.873 |  |
| Measurements: Left thyroid lobe length | p = 0.243 |  |
| Measurements: Isthmus transverse | p = 0.733 |  |
| Measurements: Isthmus anterior posterior | p = 0.366 |  |
| Optimally measured | p = 0.155 |  |
| Fairly measured | p = 0.363 |  |
| **Inaccurately measured** | **p = 0.017** |  |
| Skipped measurements | p = 0.307 |  |
| **Case 5** |  |  |
| Total procedure time | p = 0.706 |  |
| Total time with ultrasound image | p = 0.414 |  |
| **Correctly entered clinical findings** | **p = 0.008** |  |
| **Clinical findings: Thyroid nodule internal content** | **p = 0.015** |  |
| Clinical findings: Thyroid nodule shape | P = 1.000 | |
| Clinical findings: Thyroid nodule margin | p = 0.384 |  |
| **Clinical findings: Thyroid nodule calcifications** | **p = 0.041** |  |
| Clinical findings: Thyroid nodule suspected malignancy | p = 0.130 |  |
| **Acquired standard views** | **p = 0.011** |  |
| Skipped standard views | p = 0.073 |  |
| Unrecognized views | p = 0.996 |  |
| Measurements: Right thyroid lobe width | p = 0.854 |  |
| Measurements: Right thyroid lobe depth | p = 0.464 |  |
| Measurements: Right thyroid lobe length | p = 0.361 |  |
| Measurements: Left thyroid lobe width | p = 0.253 |  |
| Measurements: Left thyroid lobe depth | p = 0.900 |  |
| Measurements: Left thyroid lobe length | p = 0.911 |  |
| Measurements: Isthmus transverse | p = 0.806 |  |
| Measurements: Isthmus anterior posterior | p = 0.328 |  |
| Optimally measured | p = 0.103 |  |
| Fairly measured | p = 0.833 |  |
| Inaccurately measured | p = 0.091 |  |
| Skipped measurements | p = 0.313 |  |
| **Case 6** |  |  |
| Total procedure time | p = 0.166 |  |
| Total time with ultrasound image | p = 0.819 |  |
| Correctly entered clinical findings | p = 0.176 |  |
| Clinical findings: Thyroid nodule internal content | p = 0.052 |  |
| **Clinical findings: Thyroid nodule shape** | **p = 0.010** |  |
| Clinical findings: Thyroid nodule margin | p = 0.102 |  |
| Clinical findings: Thyroid nodule calcifications | p = 0.519 |  |
| **Clinical findings: Thyroid nodule suspected malignancy** | **p = 0.003** |  |
| **Acquired standard views** | **p = 0.003** |  |
| Skipped standard views | p = 0.161 |  |
| Unrecognized views | p = 0.773 |  |
| **Measurements: Right thyroid lobe width** | **p = 0.038** |  |
| Measurements: Right thyroid lobe depth | p = 0.424 |  |
| Measurements: Right thyroid lobe length | p = 0.305 |  |
| Measurements: Left thyroid lobe width | p = 0.183 |  |
| Measurements: Left thyroid lobe depth | p = 0.647 |  |
| Measurements: Left thyroid lobe length | p = 0.306 |  |
| Measurements: Isthmus transverse | p = 0.654 |  |
| Measurements: Isthmus anterior posterior | p = 0.690 |  |
| Optimally measured | p = 0.442 |  |
| Fairly measured | p = 0.593 |  |
| Inaccurately measured | p = 0.317 |  |
| Skipped measurements | p = 0.225 |  |
| **Case 7** |  |  |
| Total procedure time | p = 0.330 |  |
| **Total time with ultrasound image** | **p = 0.020** |  |
| Correctly entered clinical findings | p = 0.236 |  |
| **Clinical findings: Thyroid nodule internal content** | **p = 0.041** |  |
| Clinical findings: Thyroid nodule shape | p = 0.196 |  |
| Clinical findings: Thyroid nodule margin | p = 0.139 |  |
| Clinical findings: Thyroid nodule calcifications | p = 0.547 |  |
| Clinical findings: Thyroid nodule suspected malignancy | p = 0.362 |  |
| **Acquired standard views** | **p = 0.007** |  |
| Skipped standard views | p = 0.920 |  |
| Unrecognized views | p = 0.097 |  |
| Measurements: Right thyroid lobe width | p = 0.365 |  |
| Measurements: Right thyroid lobe depth | p = 0.818 |  |
| Measurements: Right thyroid lobe length | p = 0.186 |  |
| Measurements: Left thyroid lobe width | p = 0.110 |  |
| Measurements: Left thyroid lobe depth | p = 0.403 |  |
| Measurements: Left thyroid lobe length | p = 0.764 |  |
| Measurements: Isthmus transverse | p = 0.702 |  |
| Measurements: Isthmus anterior posterior | p = 0.509 |  |
| Optimally measured | p = 1.000 |  |
| Fairly measured | p = 0.507 |  |
| Inaccurately measured | p = 0.466 |  |
| Skipped measurements | p = 0.316 |  |

All metrics of the different tasks and cases. Bold font indicates p < 0.05

**Supplementary material 3**

| Thyroid nodule |
| --- |
| Present* |
| Absent |
| Internal content |
| Predominantly solid* |
| Predominantly cystic |
| Spongiform |
| Shape |
| Ovid to round* |
| Taller-than-wide |
| Irregular |
| Margin |
| Smooth* |
| Spiculated |
| Microlobulated |
| Ill-defined |
| Calcifications |
| Microcalcifications |
| Macrocalcifications |
| Rim calcifications |
| None* |
| Suspected malignancy |
| None* |
| Papillary carcinoma |
| Follicular lesion carcinoma |
| Medullary carcinoma |
| Anaplastic carcinoma |
| Thyroid lymphoma |
| Thyroid metastases |
| Cannot be determined |

Multiple choice with single answer regarding clinical findings presented by the simulator. *Most correct answer assessed by the two experts
